# The Impact of Social Determinants of Health on Outcomes Among Individuals With HIV and Heart Failure: A Literature Review

**DOI:** 10.7759/cureus.55913

**Published:** 2024-03-10

**Authors:** Pawel Borkowski, Natalia Borkowska

**Affiliations:** 1 Internal Medicine, Jacobi Medical Center/Albert Einstein College of Medicine, New York, USA; 2 Pediatrics, SPZOZ (Samodzielny Publiczny Zakład Opieki Zdrowotnej) Krotoszyn, Krotoszyn, POL

**Keywords:** better outcomes, mortality, social determinants of health (sdoh), hiv/aids, heart failure

## Abstract

This narrative review examines the complex interplay between social determinants of health (SDoH) and the outcomes for individuals living with human immunodeficiency virus (HIV) and heart failure (HF), two conditions that pose significant socioeconomic burdens globally. With millions affected by these conditions, the review delves into how socioeconomic status, education, geography, and immigration status influence health outcomes. It further explores the exacerbating roles of stigma and mental health issues, underscoring the need for comprehensive interventions and the importance of enhancing health literacy and community support. Key findings suggest that lower socioeconomic status, limited education, rural residency, and immigrant status are associated with poorer health outcomes in individuals with HIV and HF. These factors contribute to increased morbidity and mortality and decreased quality of life, highlighting the necessity of addressing SDoH to improve patient care and outcomes. There is a critical need for integrated care models that consider the medical, social, and psychological factors affecting those with HIV and HF. Strategies proposed include improving access to care, addressing socioeconomic disparities, enhancing educational efforts, and fostering community engagement. Moreover, the importance of mental healthcare integration into the management of HIV and HF is strongly advocated to improve patient outcomes. By taking a comprehensive look at the various social challenges, embracing integrated care models, and making sure everyone has fair access to healthcare services, we can make real progress in enhancing the lives of those affected by HIV and HF. This approach cannot only lower death rates but also significantly improve the quality of life for these individuals.

## Introduction and background

Human immunodeficiency virus (HIV) infection and heart failure (HF) impose a significant socioeconomic burden on affected populations and healthcare systems. The World Health Organization (WHO) estimated that at the end of 2022, 39 million people globally were living with HIV, with deaths from HIV-related complications reaching 630,000 in the same year [1]. HF, on the other hand, impacts approximately 64 million people worldwide, and in 2018, it was recorded on 379,800 death certificates in the United States [2,3].

The cost of treating both conditions is substantial, straining healthcare systems and societies. The annual cost of highly active antiretroviral therapy (HAART) ranges between $36,080 and $48,000 per patient and between $14,226 and $45,784 per patient for HF [4,5]. Individuals diagnosed with both HIV and HF face compounded health risks, leading to diminished quality of life and heightened susceptibility to further health complications [6,7]. Despite advances in medical treatments, there remains a gap in understanding and addressing the role of social determinants of health (SDoH) on the health outcomes of patients living with HIV and heart failure.

It is recognized that SDoH (such as family support, housing conditions, financial status, education, and substance abuse) plays a pivotal role in patient outcomes [8,9]. This underscores the need for holistic healthcare strategies (e.g. media campaigns, training for healthcare professionals on the social and psychological aspects of the disease, support groups, and integrated mental health services with routine care) to address the challenges posed by HIV and HF effectively. Consequently, conducting a comprehensive analysis of the impact of social challenges on mortality rates within those susceptible populations is crucial. This narrative review will explore the complex interplay between social challenges in HIV-positive and HF populations, suggesting strategies to reduce their impact on outcomes.

## Review

Addressing health disparities: the impact of socioeconomic status (SES), education, geography, immigration, stigma, and mental health on HIV and HF outcomes

The CDC defines health disparities as “avoidable differences in the incidence, prevalence, mortality, and causes of a disease and the related adverse health conditions that exist among specific population groups” [10]. This definition underscores the need for understanding health disparities. Many health disparities stem from SDoH, which include the conditions of people's birth, development, living conditions, work environments, and aging processes [11]. SDoH have been identified as significant risk factors for adverse outcomes in patients with HIV and HF [9,10]. Recognizing and understanding the influence of SDoH on health outcomes across populations are crucial for diminishing health disparities.

Socioeconomic Factors

Low SES impacts outcomes for patients with HIV and HF, with poverty being an important risk factor. Financial difficulties resulting from low income levels can lead to behaviors that increase the chances of HIV transmission and related complications [12]. For example, research by Lua et al. indicates that reduced wealth is associated with higher AIDS incidence (relative risk (RR): 1.55; 95%CI: 1.43-1.68) and mortality (RR: 1.99; 95%CI: 1.70-2.34) [8]. Furthermore, a decrease in median household income is linked to diminished survival following an HIV diagnosis [13]. Access to insurance also plays a crucial role in HIV management, as insured patients are more likely to receive comprehensive healthcare services, leading to improved life expectancy, better viral suppression, and decreased mortality rates [10]. In the context of HF, SES is similarly linked to poorer health outcomes. Supporting this, Teng et al. demonstrated that lower household income is associated with an increased one-year combined rate of all-cause mortality HF hospitalization in comparison to individuals with higher household income [14].

Education and Low Health Literacy

Individuals with higher levels of education often enjoy better health outcomes, which is attributed to their increased income and elevated social status [15]. Studies indicate an association between lower education and heightened AIDS incidence (RR: 1.46; 95%CI: 1.26-1.68) and mortality (RR: 2.76; 95%CI: 1.99-3.82) [8]. Low education level also negatively impacts HF. For instance, Barbareschi et al. found that HF patients with lower educational levels report significantly lower quality of life (QoL) than their counterparts with higher educational levels [16]. Transitioning from the direct impact of education on health outcomes, health literacy emerges as an important factor in patient empowerment and disease management. The CDC defines health literacy as “the degree to which individuals have the ability to find, understand, and use information and services to inform health-related decisions and actions for themselves and others” [17]. Nelsen et al. showed that higher knowledge about HIV is associated with better HIV viral suppression (OR:1.75, 95%CI:1.00-3.07) [18]. Notably, around 39% of HF patients display low health literacy, which leads to reduced medication adherence, deteriorated physical and mental health status, and increased rates of hospitalization [19].

Geographic Barriers

Patients with HIV and HF from rural areas face more adversities compared to their urban counterparts, as shown by several studies [20-23]. Rural residents are more likely to be diagnosed with AIDS either at the time of their HIV diagnosis or within the first year following [20]. This trend of adverse outcomes is further evidenced by the higher percentage of Black individuals receiving a late diagnosis of HIV in rural areas (25.2%) compared to those in urban and metropolitan areas (21.9% and 19.0%, respectively), underscoring the disparities faced by marginalized communities in less accessible regions [21]. In the context of HF, residing in a rural area increases the risk of mortality and correlates with fewer emergency department visits and hospitalizations [22]. Additionally, research shows that rurality compounds the risk among predominantly low-income individuals in the southeastern United States, particularly affecting women and Black men [23]. These findings reveal significant access barriers to prompt healthcare for rural populations with HIV and HF.

Immigration Status

The complexities of HIV infection and HF among migrants reveal significant health disparities compared to native-born populations. Determining the timing of HIV infection (before or after migration) is challenging, but migrant populations exhibit higher HIV prevalence rates compared to native-born individuals [24]. Similar disparities are also present within HF populations. For example, Wandell et al. analyzed a substantial cohort of immigrants in Sweden (n = 3,274,119), finding elevated HF rates among specific immigrant groups (i.e. Africa, Bosnia, Iraq) [25]. The study further highlighted that among immigrants, refugees are particularly susceptible. Moreover, Borné et al. demonstrated that being an immigrant is linked to an increased long-term risk of hospitalization for heart failure [26]. This situation is possibly exacerbated by the legal and social barriers immigrants face, which significantly restrict their access to healthcare services. Fears of deportation and ineligibility for insurance due to undocumented status or immigration conditions may deter many from seeking necessary medical care. Furthermore, immigration status often limits access to government-funded healthcare programs, placing immigrants at a more significant disadvantage.

Stigma and Mental Health

Despite the identification of HIV over four decades ago, stigma and discrimination persist, especially among marginalized groups such as homosexual men, intravenous drug users, and Black and Hispanic communities, who are most affected by the epidemic [27,28]. Conversely, the stigma associated with HF is insufficiently examined in the literature [29]. The stigma extends to mental health within these populations, where rates of psychiatric disorders are significantly higher compared to the general population. For example, Bing et al. found that nearly half of a 2,864-subject study group living with HIV screened positive for psychiatric disorders, with up to 36% suffering from major depression, up to 15.8% from generalized anxiety disorder (GAD), and up to 10.5% from panic attacks [30]. Similarly, the HF population faces a high prevalence of major depression and anxiety disorders, such as GAD, which are linked to poor medical and functional outcomes [31]. Research by Rutledge et al., reviewing 27 studies, estimated that about 21.5% of HF patients might be affected by depression [32]. In a broader analysis, Easton et al., examining 73 studies, found that 13.1% of HF patients could suffer from GAD [33]. These findings highlight the critical link between mental health disorders and HIV and HF populations. The cultural stigma surrounding mental health issues can prevent individuals from seeking necessary treatment, and mental health services may not be fully equipped to meet the specific needs and cultural contexts of these populations.

Table 1 gives a summary of the impact of various factors on HIV and HF outcomes.

**Table 1:** Impact of Health Disparities on HIV and HF Populations HIV: human immunodeficiency virus; HF: heart failure

Factor	Impact on HIV and HF Outcomes
Socioeconomic Status (SES)	Lower SES is linked to higher incidence and mortality and lower survival among the HIV population [8,13]. Insured patients with HIV have better life expectancy and decreased mortality compared to uninsured patients with HIV [10]. Similarly, lower household income is associated with increased mortality and hospitalization rates in HF patients [14].
Education and Health Literacy	Lower education is associated with higher AIDS incidence and mortality [8]. Low education level is related to low quality of life among the HF population [16]. Health literacy is crucial for patient empowerment and management of diseases, with low health literacy leading to poor outcomes among HIV and HF populations [18,19].
Geographic Barriers	Rural residents face more adversities, including higher AIDS diagnosis rates and mortality for HF patients [20-23].
Immigration Status	Migrants show higher rates of HIV and HF, with legal and social barriers significantly restricting their healthcare access [24,25]. Migrants face worse outcomes compared to native-born populations [26].
Stigma and Mental Health	Stigma and discrimination persist among the HIV population but are insufficiently examined among the HF population [27-29]. HIV and HF populations have higher rates of psychiatric disorders than the general population [30-33].

Enhancing health outcomes through peer support

The integration of peer support with routine medical care has been shown to significantly enhance outcomes for individuals living with HIV, surpassing the benefits of standard clinic follow-ups alone [34]. This underscores the critical role of a supportive social network for those affected by HIV/AIDS, highlighting its necessity for improved health [35]. Although research into the effects of social support on HF patients is still emerging, initial studies indicate an impact on outcomes such as rehospitalizations and mortality [36]. However, the extent and nature of these relationships between social support and HF outcomes remain to be fully elucidated, calling for more comprehensive research to draw definitive conclusions. This emerging evidence suggests the potential for social support mechanisms to play a significant role in the prognosis of HF, alongside its established benefits for individuals living with HIV.

Strategies for integrated care: addressing HIV and HF in vulnerable populations

Addressing the challenges of HIV and HF in vulnerable populations demands a strategy that acknowledges SDoH as a critical determinant of health outcomes. Integrated care models must prioritize SES-related disparities, offering comprehensive support beyond clinical interventions. Assisting with medication costs, healthcare costs, and insurance coverage could reduce the financial burden on patients. HAAR) for HIV and guideline-directed medical therapy (GDMT) for HF are related to improving health outcomes [37,38]. Therefore, affordable access to appropriate pharmacotherapy could decrease mortality among vulnerable populations. In addition, advocating for policies that improve housing conditions and job opportunities for people with low SES could also be crucial for long-term improvements.

Enhancing educational strategies across various settings is essential for promoting healthier behaviors and improving patient outcomes. School-based health education significantly lowers sexual risk behaviors [39]. For instance, research by Faust et al. highlights the efficacy of peer-education interventions in enhancing the absorption of HIV-related knowledge [40]. Specifically, these interventions have been shown to significantly improve risk reduction strategies, such as condom use (OR: 3.09, 95%CI: 1.83-5.22, p < 0.0001) and understanding of HIV's sexual transmission (OR: 5.86, 95%CI: 2.65-12.97, p < 0.001) as well as transmission via sharps (OR: 4.35, 95%CI: 3.21-5.90, p < 0.001). Education also provides advantages for individuals with HF. For example, educational interventions in the inpatient setting, such as a nurse-led, one-hour teaching session at hospital discharge, have successfully improved clinical outcomes, enhanced adherence to self-care, and reduced healthcare costs for patients with systolic HF [41]. By broadening the strategy, community involvement and expert counseling in managing HIV and HF could increase awareness and encourage proactive healthcare measures, potentially decreasing mortality rates and societal disease impact. Education in schools, communities, and healthcare facilities is key to improving health outcomes and reducing disease burden.

Barriers to healthcare access significantly impede effective treatment delivery, especially in the context of HIV care. For example, Lankowski et al. highlighted transportation as a considerable obstacle affecting access to care across all phases of HIV treatment [42]. To address these challenges, the authors recommend enhancing rural healthcare infrastructure, decentralizing HIV treatment services to improve accessibility, using mobile clinics to reach remote areas, utilizing point-of-care testing for prompt diagnosis and management, and offering transportation stipends to alleviate travel-related financial constraints for patients.

Hacker et al. identified five key areas to improve healthcare for immigrants: advocating for policy changes, expanding insurance options, broadening the safety net, enhancing provider training for immigrant care, and increasing educational efforts [43]. This would help undocumented immigrants and refugees access medical services without the fear of deportation, potentially improving outcomes for this at-risk group. Addressing the health disparities faced by migrants, particularly in HIV and HF management, requires a comprehensive strategy. Essential actions include collecting detailed health data for migrants to inform specific interventions and implementing policies that ensure healthcare access for all, regardless of immigration status [43]. In addition, legal and policy reforms are needed to eliminate healthcare access barriers for immigrants, such as restrictions on eligibility for government-funded health programs. Educating healthcare providers about the unique challenges faced by migrant populations will likely improve patient-provider communication and outcomes [44]. In addition, providing language-accessible services and materials ensures that all patients, regardless of their linguistic background, can access the care and information they need. To further support this, a detailed study conducted by Shamsi et al., involving a cohort of 300,918 participants, demonstrated that using translation tools can enhance the quality of healthcare delivery [45]. Integrating the above approaches with a focus on cultural competency can bridge the gap in care delivery, ensuring that immigrants and refugees receive personalized, high-quality healthcare.

To effectively address stigma, mental health issues, and discrimination in HIV and HF populations, comprehensive interventions are essential. Enhancing public awareness through media campaigns is vital, yet the short-lived impact of such campaigns highlights the necessity for ongoing public education to reduce the risks of HIV and HF [46,47]. The limited success of past public awareness campaigns, particularly among older HF patients, underscores the potential of targeted approaches such as healthcare professional training and community-led initiatives. Training healthcare professionals in the social and psychological aspects of HIV and HF can significantly improve patient care and satisfaction by fostering understanding [48]. Additionally, community-driven solutions, including support groups and advocacy efforts, are crucial for decreasing stigma. The CDC's recent engagement in community events to advance HIV prevention and care reflects an ongoing effort to identify effective strategies, though the outcomes are still unknown [49].

Peer support programs, shown to enhance outcomes for HIV patients through improved continuity of care, antiretroviral therapy (ART) adherence, and viral suppression, suggest a promising model for HF patients as well [34]. However, the effectiveness of such programs in the HF context requires further investigation. The profound link between mental health and healthcare outcomes in both HIV and HF populations necessitates integrated care models that include mental health services alongside routine medical care. This approach ensures psychiatric disorders are addressed within the overall care plan, potentially improving outcomes [50-52]. Moreover, increasing funding and resources for mental health services, including expanding insurance coverage, is imperative to support these populations adequately. In conclusion, addressing the challenges faced by HIV and HF populations requires a multifaceted approach that includes ongoing public education, healthcare professional training, community-led initiatives, peer support programs, and integrated care models, with increased support for mental health services.

## Conclusions

Managing HIV and HF requires a comprehensive approach beyond medical treatment, addressing social challenges such as SES, education, and geographic and immigration factors. The review emphasizes the roles of stigma and mental health on the outcomes. The evidence suggests that targeted strategies (such as improving access to care, addressing socioeconomic disparities, enhancing educational efforts, and fostering community engagement) are vital for reducing mortality rates and improving the quality of life for those affected by HIV and HF. Integrating mental healthcare and adopting an integrated care model to handle the medical, social, and psychological aspects are crucial. Effective management involves policies for equitable healthcare access, educational programs to enhance health literacy, and a holistic effort from policymakers, healthcare providers, communities, and patients. Additionally, longitudinal and comparative studies focusing on the efficacy of integrated care models for HIV and HF could provide insights into the most effective interventions for improving outcomes.
